# Thermal Flow Self-Assembled Anisotropic Chemically Derived Graphene Aerogels and Their Thermal Conductivity Enhancement

**DOI:** 10.3390/nano9091226

**Published:** 2019-08-29

**Authors:** Jinhui Huang, Buning Zhang, Paolo Valdiserri, Xue Huang, Guoqiang Yin, Yingde Cui

**Affiliations:** 1 School of Materials Science and Engineering, Northwestern Polytechnical University, Xi’an 710072, China; 2 Guangzhou Key Laboratory for Efficient Utilization of Agricultural Chemicals, Zhongkai University of Agriculture and Engineering, Guangzhou 510225, China; 3 Department of Industrial Engineering, Alma Mater Studiorum, Bologna University, 40136 Bologna, Italy; 4 Guangzhou Vocational College of Science and Technology, Guangzhou 510550, China

**Keywords:** Graphene Aerogel, Hydrothermal Method, Thermal Conductivity

## Abstract

In this study, we investigated the directional heating of graphene oxide (GO) dispersion to generate a temperature gradient and form a simulated “ocean current” inside the dispersion so that GO sheets could be aligned in a directional manner and then reduced and self-assembled into anisotropic reduced graphene oxide (rGO) gel. After freeze-drying and varying degrees of vacuum microwave treatment, anisotropic chemically derived graphene aerogels (AGAs) were obtained. Through performance detection and the analysis of the results, it was verified that the AGAs with certain characteristics of “ocean current” were prepared in this experiment, and its axial direction has obvious directional arrangement. After being treated by vacuum microwave for a short time (1 min.), the axial thermal conductivity of the composite materials (AGA-adsorbed paraffin) was observed to be 1.074 W/mK, and the thermal conductivity enhancement efficiency was 995%; as compared with similar thermal conductivity enhancement composites that were found in previous studies, the proposed method in this paper has the advantages of simple processing, high efficiency, and energy conservation.

## 1. Introduction

One application of graphene is to assemble two-dimensional (2D) graphene sheets into three-dimensional (3D) graphene aerogels (GAs). These GAs have ultra-high porosity and large specific surface area. When combined with the excellent properties of graphene itself, it has broad prospects in many applications, such as chemical adsorption [1,2], energy storage [3,4,5], batteries [6,7], and sensors [8,9]. Many studies have reported the use of GAs for energy storage. Yang et al. prepared GA phase-change energy storage materials with stable shape [10]. Zhong et al. studied the excellent performance of GAs in energy management [11]. Dong [12], Yang [13], Xu [14], and Wang [15] et al. studied the application of GAs in thermal energy storage and energy management through different preparation and research methods. These researchers all took advantage of GA’s high porosity, ultra-high specific surface area, and the super-hydrophobicity and ultra-high thermal conductivity of graphene itself to solve the problems of leakage, unstable circulation, supercooling, and low thermal conductivity in the phase-change energy storage process. 

GAs is generally prepared by the hydrothermal method [16] or organic sol-gel method [17], in which reduced graphene oxide (rGO) hydrogels are first prepared, and then GAs are obtained by freeze drying or supercritical drying. The GAs that were prepared with these methods are isotropic; the internal rGO sheet unit combination is desultorily, heat conduction pathways become disordered, phonon scattering is significant, and the contact thermal resistance increases, which thereby affects the heat transfer of the GAs. Therefore, some researchers have proposed anisotropic GAs, which have a relatively regular arrangement of graphene sheets, which greatly improves their thermal conductivity in one direction. At the same time, in a regular direction, GAs exhibit a high compression ratio, and the rebound rate can reach almost 100%. Therefore, they have great application advantages in heat management and high-compression materials. Peng et al. prepared directional GAs by the ice crystal template method, which greatly enhanced the longitudinal heat conduction [18]. Li et al. prepared anisotropic GAs by the directional freezing of liquid nitrogen and studying the effect of directional freezing with liquid nitrogen on the formation and thermal conductivity of GAs in detail [19]. The results showed that the thermal conductivity in the vertical direction was approximately six times that in the transverse direction after it was composited with epoxy resin, and the maximum thermal conductivity enhancement efficiency was as high as 5890%.

However, in these studies, the authors prepared the anisotropic GAs by the directional freezing (ice crystal template) method, that is, by applying a cold source to the prepared wet gel in a certain face to make the ice crystal grow in a certain direction and then extrude the graphene sheet to arrange along the crystal surface. The process of directional freezing is complicated, and the degree of reduction, speed, and size of crystal growth must be controlled. Sometimes, pre-reduction is required, followed by directional freezing, thawing, and thorough reduction. Otherwise, the binding of graphene sheets will be stronger after a high degree of reduction; this is not conducive to directional freezing, which leads to poor regularity of pores and poor control of pore size. While using 3D printing technology, Zhang et al. prepared graphene aerogels with controllable pores and macro directions. However, the aerogel features only macroscopic anisotropy, while microscopic isotropy, and the graphene sheets inside are still in disordered arrangement, not being conducive to heat transfer [20].

Ocean currents inspired this study, that is, liquid flowing from high-temperature areas to low-temperature areas, prompting the formation of “line flow” inside the liquid. In a graphene oxide (GO) dispersion, the two-dimensional structure of GO sheets, will lead them parallel to the direction of the line flow inside the dispersion (as the vertical direction resistance is too large, GO sheet will automatically rotate to the parallel direction). Therefore, after being reduced, the rGO sheets in the self-assembled rGO gel will be parallel to the direction of line flow or at an acute angle. This heat flow method is easy to operate and the reduction and orientation can be completed in one step.

## 2. Materials and Methods

### 2.1. Preparation of Anisotropic Chemically Derived Graphene Aerogels (AGAs)

GO was prepared by the improved Hummer’s [21] method. Firstly, 5 g of expanded graphite was added to a triangle flask containing 450 mL concentrated sulfuric acid and 50 mL phosphoric acid. After 30 min. of mechanical stirring at room temperature, 30 g of potassium permanganate was added slowly, followed by stirring for another 30 min.; the dispersion was then slowly heated to 50 °C for a constant-temperature reaction for 3 h. The reaction liquid was then cooled to room temperature, and then cooled below 5 °C in an ice bath. Subsequently, 600 mL of ice water was slowly added; the dispersion temperature was maintained below 5 °C during the adding process. Finally, 30 mL of 30% hydrogen peroxide was added. The GO samples were obtained after centrifugal washing and drying.

Next, 0.1 g of GO was added into a glass bottle, and 50 ml of 30% ethanol solution was added to obtain the GO dispersion with a concentration of 2 mg/ml. After the dispersion was placed in an ultrasonic bath for 1 h, 500 µL of concentrated ammonia water was added and stirred evenly; then, the glass bottle was placed into the hydrothermal reduction device. In the device, the temperature was programmed to rise from 30 °C to 130 °C within 8 h, and then kept at that temperature for 1 h to obtain wet gel. Next, it was soaked in deionized water for 48 h, during which the water was changed six times. After that, it was frozen at −45 °C for 3 h, and then freeze-dried for 48 h. Finally, it was microwaved under vacuum to obtain AGA. 

**Figure 1:**
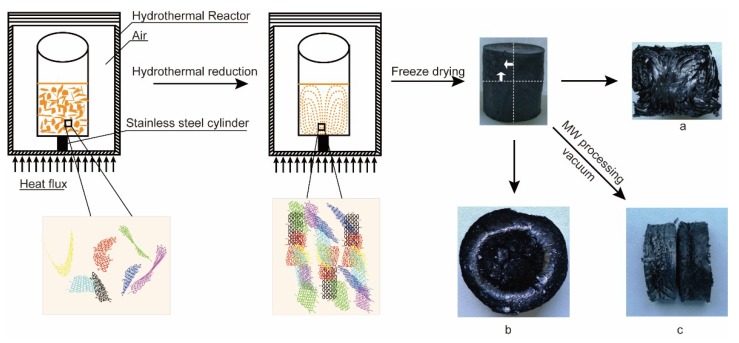
Schematic diagram of Anisotropic Chemically Derived Graphene Aerogels (AGAs) preparation steps. A digital image of the AGA’s longitudinal section (**a**) and a digital image of the AGA’s cross section (**b**); a digital image of the AGA before and after microwave treatment (**c**).

Figure 1 shows the preparation process and device. In this experiment, samples with different concentration and microwave treatment time were prepared and labeled CxWy (shown in Table 1), where x is the GO concentration (mg/mL) and y is the microwave treatment time (min.).

Some AGA samples were immersed into heated and melted paraffin wax (Guangzhou Reagent, #52), absorbed by vacuum for 30 min., and then taken out and naturally cooled to obtain AGA/paraffin composite material, denoted as PCxWy.

### 2.2. Performance and Structure Characterization

Thermogravimetric analysis (TGA) of the AGA samples was performed while using a synchronous thermal analyzer (Mettler Toledo TGA 2). Approximately 5 mg of sample was put into an alumina crucible, and then heated from 50 °C to 300 °C or 1080 °C at a rate of 10 °C/min. under a nitrogen rate of 50 mL/min. 

Thermal performance analysis of samples were measured by a Differential scanning calorimeter (DSC, TA Q2000) at a scanning rate of 10 °C min^−1^.

An ESCALAB 250Xi (Thermo Fisher Scientific) was used for X-ray electron spectroscopy (XPS) detection, with the following parameters: Monochrome Al Ka (hv = 1486.6 eV), power 150 W, 500 µm beam speckle. The binding energy was calibrated at C1s 284.8. Xpspeak41 software was used for peak fitting, with 20% Gaussian and 80% Lorentzian fixed and Shirley background mode being adopted.

The microstructures of the AGA samples were analyzed by scanning electron microscopy (SEM) with a Carl Zeiss EVO18 microscope (Carl Zeiss, Jena, Germany). The sample was placed on conductive adhesive for observation without gold spraying.

Raman spectroscopy was carried out by a microscope confocal Raman spectrometer (Horiba Jobin Yvon LabRAM HR800) with a wavelength of 633 nm. The in-plane crystal size of the sample was calculated by the following formula [22]

La(nm) = 2.4 × 10^−10^ × λ_laser_^4^ (I_D_/I_G_)^−1^ (1)

where λ is the Raman detection wavelength.

X-ray diffraction (XRD) testing was performed by a Brucker D8 Advance (Germany), with the following conditions: Voltage 40 kV, current 40 mA, step length 0.02°, test speed 0.1 s/step, copper target, and ray wavelength 0.15418 nm.

The average crystal size of the samples was calculated by the Scherrer formula [23]:

Lc = 0.89λ/(B_1/2_(2θ) × cos(θ)) (2)

where λ and B_1/2_(2θ) are the X-ray diffraction wavelength and the full width at half maximum in radian units, respectively.

The thermal conductivity of AGA and paraffin composite materials was measured while using the transient hot wire method on a thermal conductivity meter (XIATECH TC3100, China).

The enhancement efficiency in thermal conductivity of the paraffin composites can be calculated by

*η* = (*K* − *K_m_*) / (100 × *V* × *K_m_*) × 100% (3)

where *η* is the thermal conductivity enhancement efficiency; *K_m_* and *K* are the thermal conductivities of paraffin and its composite, respectively; and, *V* is the volume content of AGA in the composite.

## 3. Results and Discussion

**Figure 2:**
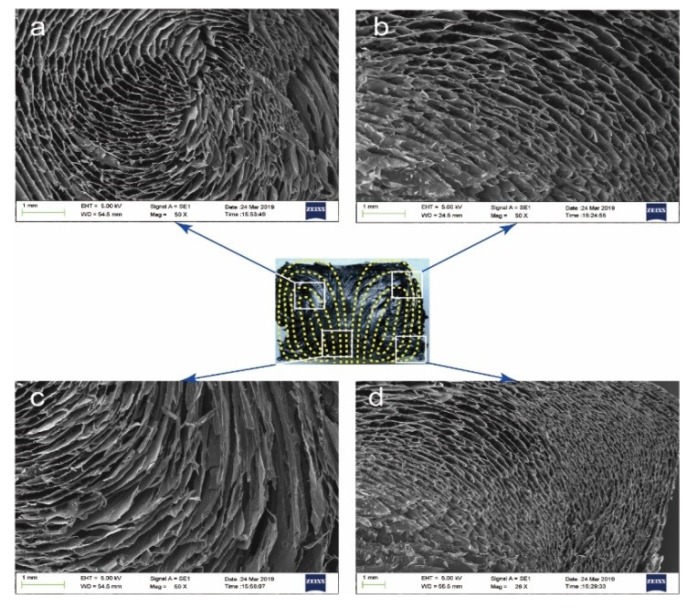
Scanning electron microscopy (SEM) images of different heat flow locations. (**a**–**d**) are electron microscope images of different positions in the longitudinal section of AGA.

**Figure 3:**
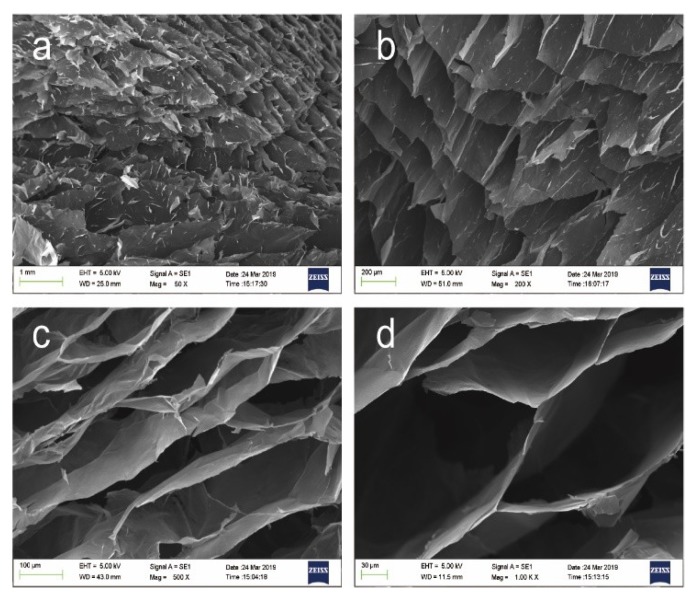
SEM images of local magnification of AGA. (**a**,**b**) are the side views of the AGA, and (**c**,**d**) are the top views of the AGA

In this study, directional AGA samples were prepared by the heat flow method, their physical and chemical properties were tested, and the thermal conductivity enhancement of the composites was studied after the adsorption of paraffin. Figure 1 illustrates the preparation steps of the AGA samples. A glass bottle containing GO dispersion was supported by a stainless steel cylinder in a reactor, which was placed in an oven for hydrothermal reduction. As shown in Figure 1a, the internal structure of the AGA adopts the shape of ocean currents and, in Figure 1b, an inner ring is observed. This inner loop occurs because the rGO sheets are parallel to the bottom at the point where the heat flow rotates (i.e., the interface where the heat flow changes from rising to falling). After vacuum microwave treatment, AGA shows a noticeable metallic luster, as shown in Figure 1c (left). A directional AGA was found in the SEM image in Figure 2, similar to the heat flow direction. It is obvious that the heat flow direction is highly similar to the direction of AGA formation. From the side view of the aerogel (Figure 3a,b), it can be observed that the AGA has a lamellar structure, which confirms that the rGO sheet is parallel to the direction of heat flow. rGO is laminated and aggregated on the isofluidics and crosslinked between non-isofluidics to form gels. As seen in Figure 3c,d, there is indeed a large number of rGO sheets crosslinked between the two layers, which result in a bridge structure, which makes the connections between the layers close and forms a relatively dense network structure.

**Figure 4:**
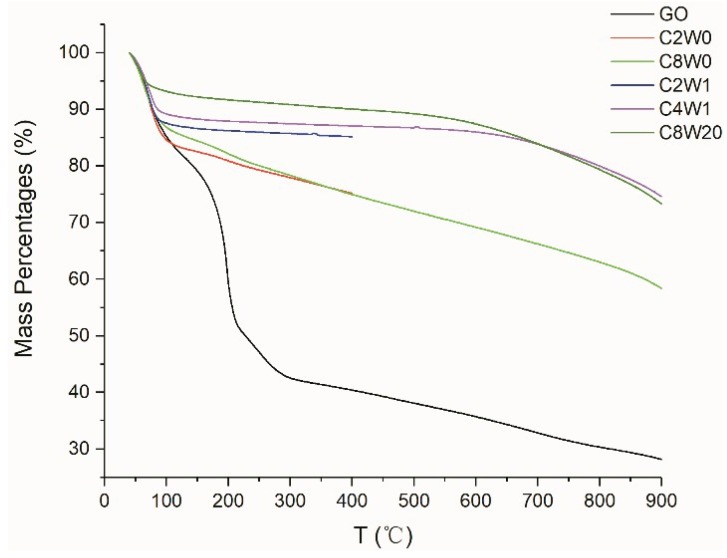
Thermogravimetric analysis (TGA) curves of different samples.

TGA testing was carried out to further reveal the thermal stability of the aerogel; the results in Figure 4 show that GO experienced two significant mass loss conditions. The first occurred near 100 °C, which was the mass loss of adsorbed water, and the second occurred near 200 °C, which was the dissociation of oxygen-containing groups; this was the major mass loss condition of GO. After hydrothermal reduction, most of the oxygen-containing groups have been reduced and eliminated, and no obvious weight loss can be seen near 200 °C. The weight loss of the samples that were treated by microwave at 100 °C is less than that of the samples not treated by microwave, which indicates that the absorbed water content is reduced and the hydrophilic groups (such as oxygen-containing groups and nitrogen-containing groups) are reduced. In addition, from 200 °C to 600 °C, the samples that were treated by microwave have little or no weight loss, which further indicates the decrease of hydrophilic groups. This implies that the oxygen-containing or nitrogen-containing groups that fail to be eliminated after hydrothermal reduction lose some weight after microwave treatment, which was also verified in the subsequent XPS analysis. After 1 min. of microwave treatment, the mass loss of the high-concentration samples at 100 °C is less than that of the low-concentration samples, possibly because the pore wall of the high-concentration samples is thicker and the specific surface area is smaller than that of the low-concentration samples (it is difficult for moisture to enter adjacent layers and the adsorbed moisture is mainly concentrated in the pore surface or capillary of the aerogel). Therefore, the area of adsorbed water is smaller than that of the low-concentration samples, which results in less weight loss of water. However, as the temperature increases, the microwave-treated samples begin to lose weight again near 600 °C, which due to the decomposition of the remaining oxygen-containing and nitrogen-containing groups after hydrothermal reduction and microwave treatment (it was verified in the subsequent XPS analysis, that is, after 20 min. of microwave treatment, AGA still contained 7. 5 *wt*% O and 8.5 *wt*% N). 

**Figure 5:**
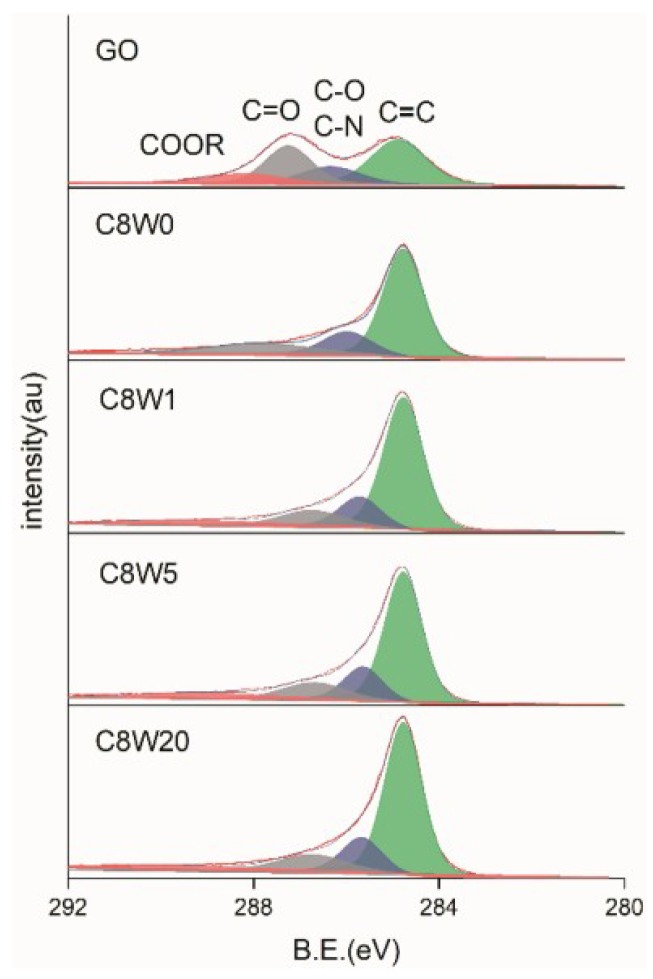
C1s X-ray electron spectroscopy (XPS) fitting curves for different microwave processing durations.

**Figure 6:**
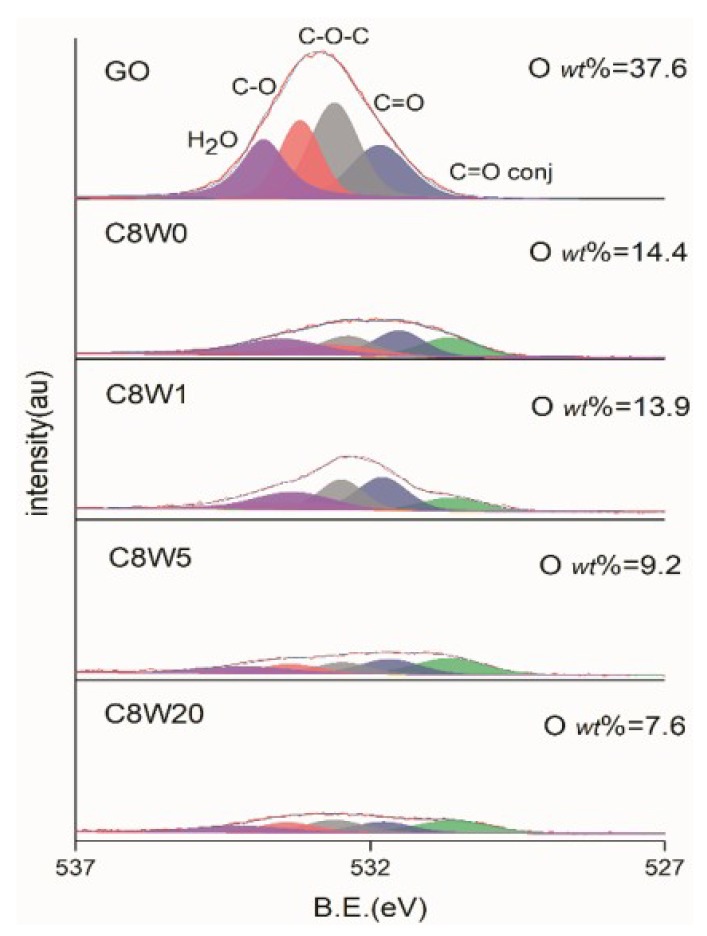
O1s XPS fitting curves of different microwave processing durations.

**Figure 7:**
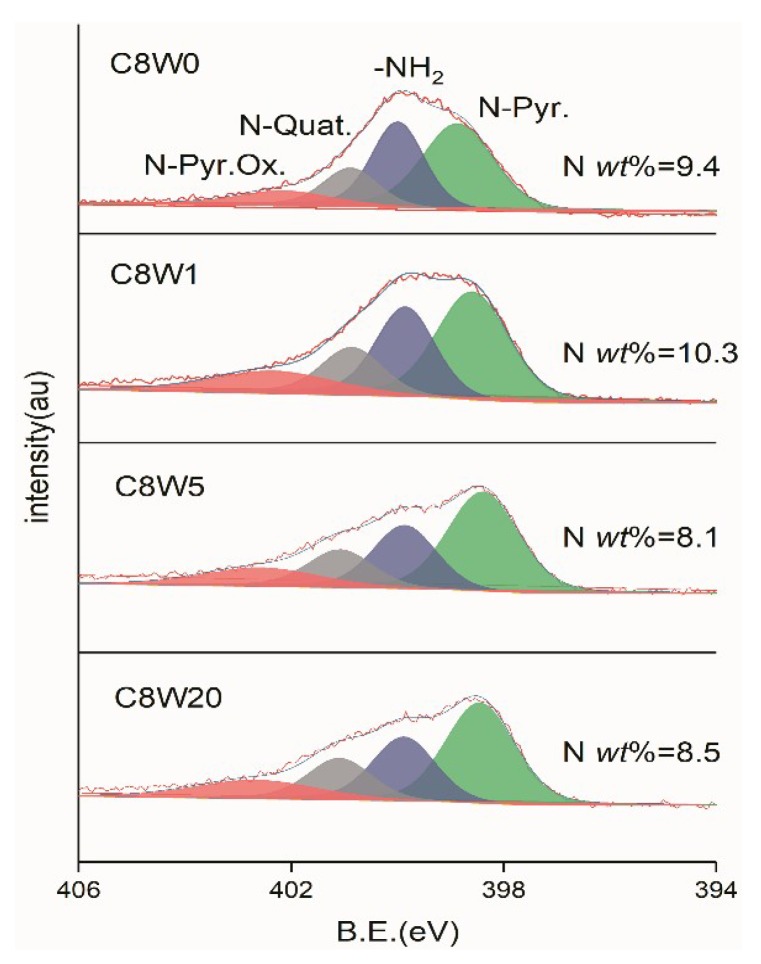
N1s XPS fitting curves of different microwave processing durations.

The dissociation and content of oxygen-containing groups in the AGA samples were further studied through XPS detection and analysis. Table 2 shows the energy spectrum peaks of C1s, O1s, and N1s in different groups reported in the literature. As can be seen from the C1s fitting curve in Figure 5, after hydrothermal reduction and microwave treatment, sp2 carbon (C=C) content increased, C=O significantly decreased, and almost all COOR was reduced, leaving only a small amount of C=O and C-O (C-N). After microwave treatment, the peak position shifted to the right, which indicated that C=O and C-O were decomposed, while C=O conj and C-N were not decomposed. This result is verified with Figure 6 and Figure 7. After microwave treatment for 20 min., C=O was significantly reduced, but C=O conj bond increased, with an O content of 7.6% and an N content of 8.5%. This is corroborated by the O1s fitting curve in Figure 6, except that the content of C=O conj bond increases in the sample after reduction (consistent with the research results in the literature [24], which is attributed to the C=O bond and C=C conjugate); other oxygen-containing groups all significantly decrease to different degrees. However, the moisture content of the samples without microwave treatment and treatment for a short time (1 min.) is still high, which verifies the mass loss near 100 °C observed in the TGA results. 

Figure 7 shows the fitting curve of N1s. It can be seen from the figure that the content of N can be reduced to some extent by microwave treatment. From the peak shape change, this is mainly due to the dissociation of -NH_2_. The difference in N content without microwave treatment and after 1 min. of treatment might be caused by the difference in local areas of XPS scanning.

**Figure 8:**
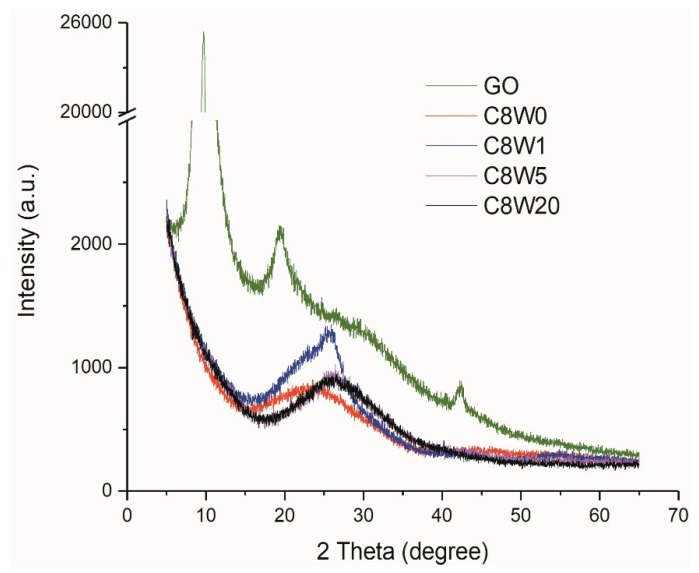
XRD curves of different samples.

The physical properties of the AGA samples were characterized by XRD. From the XRD curves shown in Figure 8, it can be seen that the GO characteristic diffraction angle appears at 9.7°. This is due to oxidation-intercalation, and the adsorption of water, which expands the layer spacing, which shifts the diffraction angle to the left. After hydrothermal reduction, the peak moves to the right and then appears near 24.4 °, and the peak at 9.7° almost disappears [29]. XPS analyses shown that there was still approximately 14.4 *wt*% (C8W0) oxygen content after hydrothermal reduction. These oxygen atoms form covalent bonds with carbon atoms, which increase the graphite lattice along the c-axis, and reduce the diffraction angle of the reduced graphite. However, after microwave treatment, oxygen-containing groups further decompose and leave [30], which makes the lamination between layers more compact; its diffraction angle of 26.1° is quite close to that of natural graphite (26.5°).

**Figure 9:**
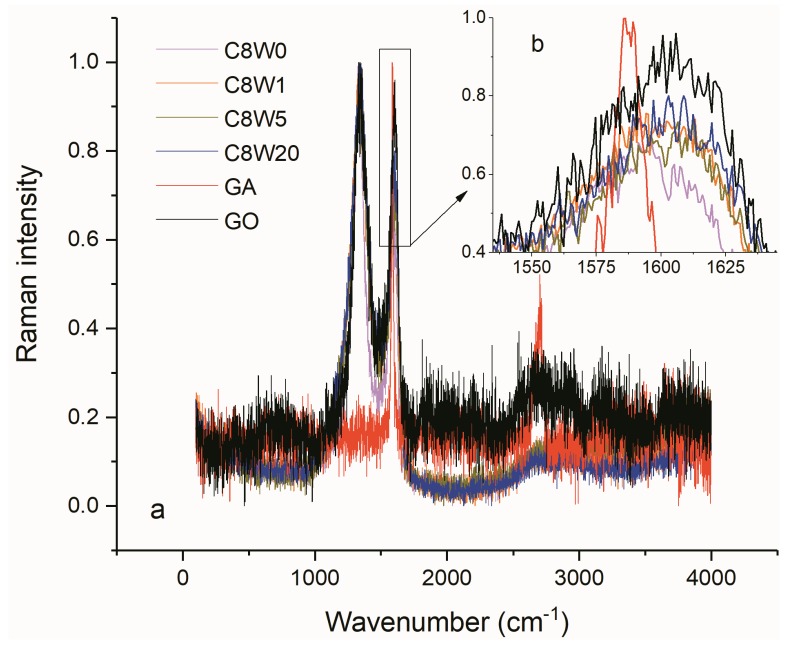
Raman normalization curves of different samples (**a**) and the amplification view of peak G (**b**).

Figure 9 shows the normalized Raman curves for different samples. As shown in the figure, the disordered vibration peak of graphene, the D peak, appeared near 1330 cm^−1^; it was caused by the lattice vibration leaving the center of Brillouin region and it is used to characterize the structural defects of the graphene samples. The vibration peak of sp2 in-plane, the G peak, appeared near 1600 cm^−1^, which represents a large deviation from 1580 cm^−1^ of graphene. As can be seen from Figure 9b, with the increase in microwave processing time, the intensity of the G peak does not significantly increase, and I_D_/I_G_ does not show a significant decreasing trend. Although a longer microwave treatment time causes a higher reduction degree (lower oxygen content), at the same time more fragments are generated, which leads to an increase in the boundary effect and an increase in the intensity of the D peak [31,32]. In-plane crystal size La [33] and average crystal size Lc (Table 3) also decrease with the extension of microwave processing time, which also confirms that a longer microwave processing time causes a higher reduction degree and it generates more fragments at the same time.

**Figure 10:**
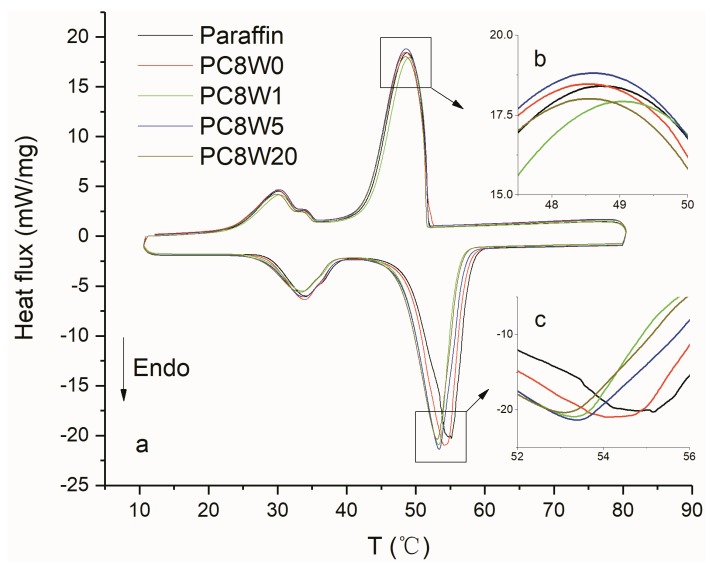
DSC curves of different AGA/paraffin composites (**a**); (**b**,**c**) are the amplification view of the exothermic peaks and endothermic peaks, respectively.

The thermal properties of AGA adsorbed paraffin were studied by DSC and thermal conductivity testing. According to the DSC curve in Figure 10, the addition of AGA changes the phase transition temperature of paraffin. The addition of AGA reduces the supercooling of paraffin by 1.9 °C at most, as shown by the changes in the subcooling degree of AGA/paraffin composite materials in Table 4. AGA after microwave treatment can better reduce the subcooling degree of paraffin, but a longer treatment time does not improve the effect of reducing the subcooling degree. This is because the thermal conductivity of graphene has a close relationship with the in-plane crystal size La. A smaller La results in a smaller thermal conductivity, and a higher I_D_/I_G_ value indicates that the high boundary effect and defects lead to high phonon scattering and high thermal resistance, which thus reduces the thermal conductivity of AGA. The results and analysis of Raman spectra and XRD indicate that the extension of microwave treatment time does not significantly reduce the I_D_/I_G_ value, but it does decrease the crystal size La. As a result, the supercooling degree of PC8W1 is relatively low, but there is no significant difference between PC8W5 and PC8W20. Usually, a longer microwave processing time is associated with higher temperature and the annealing effect, and the AGA graphite structure can be better restored, but perhaps in this experiment, the laboratory-made vacuum microwave processing equipment caused microwave energy losses, which resulted in insufficiently high temperatures (in the experiments, only a flash was observed, rather than a red-hot phenomenon), and the vacancies and defects of the rGO could not be repaired. Li et al. found that the oxygen-containing groups could be completely removed only after annealing above 1500 °C, upon which the repairs to vacancies and defects could be observed. It may also be that the reduction at a lower temperature, which has the positive effect on thermal conductivity enhancement of sp2 domain (generated by rGO), is weaker than the negative effect of phonon scattering that is caused by boundaries or stacks. Fan et al. also found that the thermal conductivity of high-density AGAs after annealing at 450 °C is lower than that without annealing [34].

**Figure 11:**
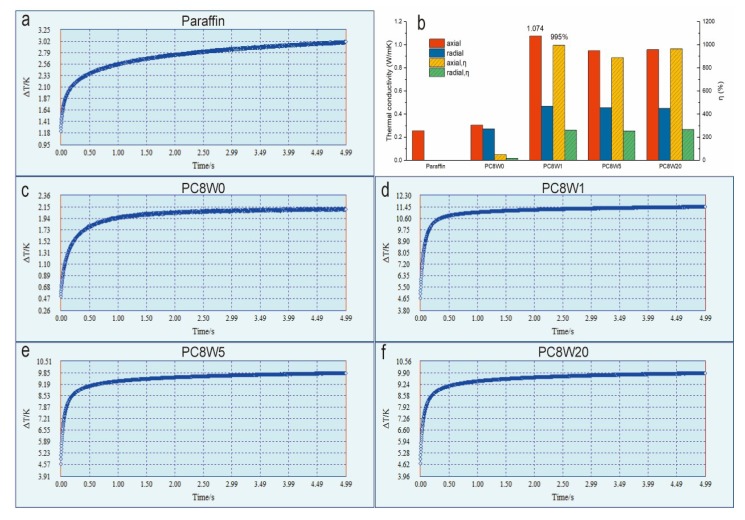
(**a**,**c**–**f**)are the curves of temperature difference with time during the thermal conductivity test of AGA/paraffin composite materials, and (**b**) is the thermal conductivity (axial).

As can be seen from the axial-direction temperature-change curves over time in Figure 11a,c–f, the AGA/paraffin composite materials reach the equilibrium temperature in a relatively short time, especially when treated by microwave. This indicates that AGAs accelerate the diffusion of heat, and the thermal conductivity is improved. Figure 11b shows the axial and radial thermal conductivity and thermal conductivity enhancement efficiency of different samples. Among them, PC8W0’s thermal conductivity enhancement effect is not obvious, because a considerable part of oxygen-containing groups (oxygen content up to 14.4 *wt*%) and nitrogen-containing groups remain, although most oxygen-containing groups can be removed by a hydrothermal reduction reaction. The presence of these groups will seriously affect the integrity of the graphite structure, thereby affecting the thermal conductivity effect [30,35,36]. For the AGAs that were treated by microwave, the contents of both oxygen and nitrogen-containing groups were significantly reduced. Moreover, Raman and XRD analysis showed that the integrity of the AGA graphite structure was improved, so the thermal conductivity significantly increased. The maximum axial thermal conductivity was 1.074 W/mK (0.32 vol %), and the thermal conductivity enhancement efficiency was 995%. However, the extension of microwave treatment time causes an improvement in the reduction degree, which leads to an increase in rGO fragments and interfacial thermal resistance. Therefore, the improvement of thermal conductivity is not obvious with the extension of microwave treatment time. It can be seen from Figure 11b that the anisotropic AGA/paraffin composites that were prepared by the heat flow method have significant differences in thermal conductivity between the axial and radial directions. This is because the directionally arranged rGO sheets in the axial direction have a large contact area, which can reduce the contact thermal resistance and form a more perfect thermal conduction path. However, the radial contact points and areas are smaller than those in the axial direction, which leads to a loose interconnection network and increases the contact thermal resistance [37].

Many studies have reported that graphene enhances thermal conductivity, and many factors that affect the thermal conductivity of graphene composites have also been reported. In addition to the influence of the quality of the graphene itself (such as size [38], thickness, defects, and shape [39]) and the macroscopic structure of graphene (such as the presence of a 3D network structure, density, and pore characteristics), it is also related to the composite materials. Different levels of compatibility between different composites (e.g., paraffin, resin, and silicone) and graphene interfaces also lead to different thermal resistance at the graphene-composite interface, which thus leads to different degrees of enhanced thermal conductivity. From the results of references [40] and [11], as shown in Table 5, one is adsorbent paraffin, and the other is adsorbent octadecanoic acid; the GAs preparation and treatment methods are the same, but the thermal conductivity enhancement efficiency is quite different (the former is 1144%, and the latter is 66.7%). Of course, there are other reasons mentioned previously. Table 5 compares the results of several recent studies on the thermal conductivity enhancement of graphene paraffin composites. It is clear that the thermal conductivity enhancement efficiency of AGAs that were prepared by the heat flow method in this study is quite good, but the effect is not as good as that of reference [40], which was possibly because the latter is well repaired after annealing. The axial thermal conductivity enhancement efficiency of reference [19] is as high as 5890%. The main reason is that the defects and vacancies of graphene can be well repaired by annealing at a high temperature for a long time to obtain a high-quality graphene. Moreover, an anisotropic GA can be obtained after directional freezing, to reduce the thermal resistance in the axial direction. 

As shown in Table 5, the preparation of graphene composites with high thermal conductivity enhancement efficiency can be considered from the following aspects: 1. It is necessary to have a good 3D thermal conductivity network (such as aerogel), or to increase the content, so that graphene fillers can connect with each other to conduct heat. 2. High-quality graphene is required, such as that processed by high-temperature annealing, mechanical exfoliation, or chemical vapor deposition (CVD) preparation. 3. Anisotropic GAs should be prepared to form a regular arrangement in a certain direction and reduce the thermal resistance of contact between graphene sheets. Obviously, preparing AGAs by the heat flow method that was proposed in this paper is a simple process that does not require additional directional freezing. Furthermore, instead of high-temperature annealing, with a simple microwave treatment, efficient and energy-conserving, it can also obtain AGAs with high thermal conductivity enhancement efficiency.

## 4. Conclusions

Higher-quality anisotropic AGAs were prepared by the heat flow method with vacuum microwave treatment to significantly reduce the oxygen content and nitrogen content. After the vacuum microwave treatment of AGA for a short time (1 min.), the axial thermal conductivity of AGA/paraffin composite material reached 1.074 W/mK and the thermal conductivity enhancement efficiency reached 995% when the content was 0.32 vol%. However, XRD and Raman analysis show that the apparent quality of the rGO does not significantly improve with the extension of microwave time. The average in-plane crystal size La decreases, and the decrease in I_D_/I_G_ is not obvious. This may attribute to the vacuum microwave device, attenuating the microwave to a certain extent, and lowering the sample temperature, which results in an unsatisfactory repair effect of rGO vacancies and defects. Therefore, the method of preparing AGAs by the heat flow method that is proposed in this paper has the advantages of simple processing, high efficiency, and energy conservation. The high thermal conductivity enhancement efficiency of the AGAs lends them to a high application value in phase-change energy storage, thermal energy management, and other fields.

## Figures and Tables

**Table 1:** Sample Numbers for different graphene oxide (GO) concentrations and microwave treatment times.

Sample Number	GO Concentration (mg/mL)	Microwave Processing Time (min)
C2W0	2	0
C2W1	2	1
C4W1	4	1
C8W0	8	0
C8W1	8	1
C8W5	8	5
C8W20	8	20

**Table 2:** Assignments of the O1s, C1s, and N1s peaks according to the literatures [25,26,27].

C1s		O1s		N1s	
Species	Range(eV)	Species	Range(eV)	Species	Range(eV)
C=C	284.6	C=O,conj [28]	530.7	N-Pyr.	398.5
C-O,C-N	286.3	C=O	531.5	-NH_2_	400.0
C=O	287.2	C-O-C	532.6	N-Quat.	401.2
COOR	288.9	C-O	533.5	N-Pyr. Ox.	402.5
		H_2_O	534.0		

**Table 3:** Lc and La of AGAs with different microwave treatment durations.

Samples	Lc (nm)	La (nm)
C8W1	4.0	31.0
C8W5	2.4	30.4
C8W20	1.4	30.0

**Table 4:** Subcooling degree of AGA/paraffin composite materials.

Samples	Paraffin	PC8W0	PC8W1	PC8W5	PC8W20
Supercooling degree (°C)	6.1	5.5	4.2	4.8	4.5
Reduce degree (°C)	-	0.6	1.9	1.3	1.6

**Table 5:** Comparison of graphene-enhanced heat conduction reported in the literature with that of this study.

Samples	Loading	*k*, *W/(mK)*	Preparation Methods	*η*,year
Graphene/Paraffin	3 *wt*%	0.274	rGO	27% [41]
GAs/Paraffin	2 *wt*%	2.111	rGO + Annealing (1000 °C, Ar,30 min.)	1144% [40]
Graphene foam /Paraffin	2.54 *vol*%	1.82	rGO + CVD	226% [42]
Graphene/Paraffin	0.4 *vol*%	0.418	rGO	586% [43]
Graphene/Paraffin	8 *wt*%	1.73	- ^a^	162% [44]
GO/CNTs/Paraffin	1.314 *wt*%	0.813	rGO + CNT	605% [45]
AGAs/Paraffin	0.32 *vol*%	1.074	rGO + Wave(800 W,vacuum,1 min.), Anisotropy	995%, this work
GAs/Epoxy resin	0.75 *vol*%	6.57	rGO + Annealing (2800 °C, Ar, 2 *h*), Anisotropy	5890% [19]
GAs/Octadecanoic acid	20 *vol*%	2.635	rGO + Annealing (1000 °C, Ar, 30 min.)	66.7% [11]

Note: ^a^, the author didn’t provide the preparation method of graphene.
